# The Role of Regulatory T Cells in the Onset and Progression of Primary Sjögren’s Syndrome

**DOI:** 10.3390/cells12101359

**Published:** 2023-05-10

**Authors:** Varvara G. Blinova, Vladimir I. Vasilyev, Ekaterina B. Rodionova, Dmitry D. Zhdanov

**Affiliations:** 1 Laboratory of Medical Biotechnology, Institute of Biomedical Chemistry, Pogodinskaya St. 10/8, 119121 Moscow, Russia; varya.blinova@list.ru; 2 Joint and Heart Treatment Center, Nizhnyaya Krasnoselskaya St. 4, 107140 Moscow, Russia; nsshornikova@gmail.com; 3 Medical Center Ltd., Timura Frunze St. 15/1, 119021 Moscow, Russia; kate.76@mail.ru

**Keywords:** regulatory T cells, primary Sjögren’s syndrome, FoxP3, Treg-based therapy

## Abstract

Regulatory T cells (Tregs) play a key role in maintaining immune balance and regulating the loss of self-tolerance mechanisms in various autoimmune diseases, including primary Sjögren’s syndrome (pSS). With the development of pSS primarily in the exocrine glands, lymphocytic infiltration occurs in the early stages, mainly due to activated CD4^+^ T cells. Subsequently, in the absence of rational therapy, patients develop ectopic lymphoid structures and lymphomas. While the suppression of autoactivated CD4^+^ T cells is involved in the pathological process, the main role belongs to Tregs, making them a target for research and possible regenerative therapy. However, the available information about their role in the onset and progression of this disease seems unsystematized and, in certain aspects, controversial. In our review, we aimed to organize the data on the role of Tregs in the pathogenesis of pSS, as well as to discuss possible strategies of cell therapy for this disease. This review provides information on the differentiation, activation, and suppressive functions of Tregs and the role of the FoxP3 protein in these processes. It also highlights data on various subpopulations of Tregs in pSS, their proportion in the peripheral blood and minor salivary glands of patients as well as their role in the development of ectopic lymphoid structures. Our data emphasize the need for further research on Tregs and highlight their potential use as a cell-based therapy.

## 1. Introduction

Primary Sjögren’s syndrome (pSS) is a systemic autoimmune and lymphoproliferative disease characterized by the appearance of uncontrolled lymphoplasmacytic infiltration in glandular tissues with subsequent development of infiltration in the lungs, kidneys, vascular walls, and other organs. The primary target organs in pSS are exocrine glands (such as salivary, lacrimal, and sweat glands [1,2]), which are glands of the gastrointestinal and respiratory systems, which confirms the conception of generalized autoimmune epithelitis in this disease. In the absence of therapy, patients can develop systemic manifestations such as various types of lesions in the joints, blood vessels (cryoglobulinemic and hypergammaglobulinemic purpura), lungs, kidneys, and reticuloendothelial (regional and generalized lymphadenopathy, splenomegaly, and hepatomegaly), peripheral, and central nervous system, which can lead to a significant decrease in the quality and life expectancy of the patients [3,4]. According to studies, gastrointestinal involvement (dysphagia) can be observed in up to 80% of patients, and arthralgia is reported to be present in up to 75% of patients [5]. The earliest clinical manifestations of the disease are dry syndrome (dry mouth, eyes, and nasopharynx), recurrent sialadenitis, joint damage (arthralgia, non-erosive arthritis, and morning stiffness), photodermatosis, and various types of hemorrhagic eruptions. Immunological symptoms such as hypergammaglobulinemia, the detection of rheumatoid factor (RF), antinuclear factor (ANF), Sjögren’s syndrome antigen A (Ro/SSA) and Sjögren’s syndrome antigen B (La/SSB) antibodies, and anticentromeric antibodies (ACAs), can be determined many years before the development of clinical manifestations of the disease. Additionally, secondary Sjögren’s syndrome (sSS) can be distinguished, which is associated with lesions in the secreting epithelial glands in patients with other rheumatological conditions (rheumatoid arthritis, systemic lupus erythematosus, systemic scleroderma, etc.), hepato-biliary (autoimmune hepatitis, primary biliary cholangitis, and primary sclerosing cholangitis), cross-syndromes, and Hashimoto’s autoimmune thyroiditis. Often, patients meet the criteria for 2–3 autoimmune diseases, and then, it is more correct to consider them as a combination of diseases, for example, pSS + rheumatoid arthritis + primary biliary cirrhosis of the liver, and not sSS. pSS and sSS are the most common autoimmune rheumatic diseases and affect 1–1.5% of the US population [3]. In 15% of patients, the disease debuts in childhood, and in 70% under the age of 50 years. pSS is more common in females, and the ratio to males ranges from 9:1 to 25:1 [3,4]. This phenomenon can be explained by several reasons. First, the immune systems of women are characterized by a higher background level of serum immunoglobulins and, consequently, more pronounced humoral immune responses [6]. This fact is consistent with the generally accepted hypothesis that the immune systems of women react more actively to various antigens, which results in the greater prevalence and aggressiveness of autoimmune diseases in women [7,8]. Second, the selective prevalence of pSS among women is also associated with the various effects of sex hormones on the immune system. It was demonstrated that estrogens can play a great role in the development and progression of pSS [9]. However, the general effect depends on the form of the estrogen, its concentration, the relevant receptor signaling pathways, and age. In fact, it was shown that physiological (low) levels of estrogen can enhance the pro-inflammatory capacity of human and murine macrophages and monocytes, whereas supraphysiological levels can lead to the opposite effect [10]. In addition, reduced levels of estrogen with menopause could decrease the protective effect of these hormones by increasing inflammation and by reducing its proliferative effects on glandular cells leading to increased apoptosis and autoantibody production [11]. Along with that, it was reported that estrogen receptors in the salivary gland epithelial cells of SS patients have reduced responsiveness to estrogen [12]. In the development of pSS, it is more likely that estrogens implement their functions in the context of genetic factors and other environmental stimuli. In these circumstances, they contribute to the polyclonal activation of B lymphocytes, the formation of autoantibodies, and an increase in the level of prolactin, which aggravates the severity of the disease [13]. Androgens, on the contrary, act as an inhibitory factor, reducing the severity of immunopathological manifestations in pSS [14]. Currently, the standard for determining the degree of pSS activity is the EULAR Sjögren’s Syndrome (SS) Disease Activity Index (ESSDAI). Additionally, in some studies, mild (SS-I), intermediate (SS-II), and severe (SS-III) groups of minor salivary glands (MSGs) in biopsy specimens are distinguished, which is based on the severity of infiltration of these glands [15]. In the absence of early diagnosis and treatment of the disease, due to inadequate activation and proliferation of T and B lymphocytes, ectopic lymphoid structures are formed in epithelial tissues. They are initially characterized by the synthesis of polyclonal and later oligoclonal and monoclonal immunoglobulins (Igs) with development in 5–11% of predominantly different variants of B-cell lymphomas [3,4]. Lymphoepithelial lesions (LELs) with ectopic lymphoid structures are found in 90–100% of patients with enlarged major salivary glands in their biopsy specimens, whereas their presence in MSGs is significantly lower [16]. MALT lymphomas (from mucosal-associated lymphoid tissue) in the salivary/lacrimal glands, lungs, stomach, and thymus and diffuse large B-cell lymphomas (DLBCLs) affecting the lymph nodes, bone marrow, salivary/lacrimal glands, Pirogov–Waldeyer rings, etc., are the most common lymphoma subtypes in pSS [17], being the hallmark of this disease. Most DLBCLs in pSS/sSS are transformed from MALT lymphomas in the absence of diagnosis and treatment of significantly enlarged major salivary/lacrimal glands and focal lung infiltrates [18]. In the absence of anti-lymphoproliferative therapy, MALT lymphomas develop in the first 10 years of the disease (the median disease duration from pSS onset to MALT lymphoma diagnosis is approximately 7 years) [5], while DLBCLs develop 17–20 years after the onset of the disease [17]. Given the high incidence of lymphomas in pSS, the disease is considered to be both autoimmune and lymphoproliferative and is a natural model for studying the development from autoimmunity to lymphoproliferation [3,4].

The main factor in the onset of pSS and the development of lymphomas in this disease is considered to be the autoactivation of immune cells. At the same time, the main cells of the immune system that suppress the action of activated lymphocytes are regulatory T cells (Tregs). A decrease in their number in the peripheral blood and/or insufficiency of functional activity is also associated with the development and progression of pathological processes in patients with pSS [15]. While the main demographic, epidemiological, clinical, and laboratory studies are well covered in the main monographs and latest reviews [3,4], many pathogenetic, generodionovatic, and molecular mechanisms in the development of these pathological conditions, their transition to lymphoproliferation, and therapeutic approaches are not well understood and require further research.

The purpose of this review is to organize and arrange the data on the role of Tregs in the pathogenesis of pSS, as well as to consider possible strategies of Treg-based therapy for this disease.

## 2. Immunopathogenesis of pSS

**Figure 1:**
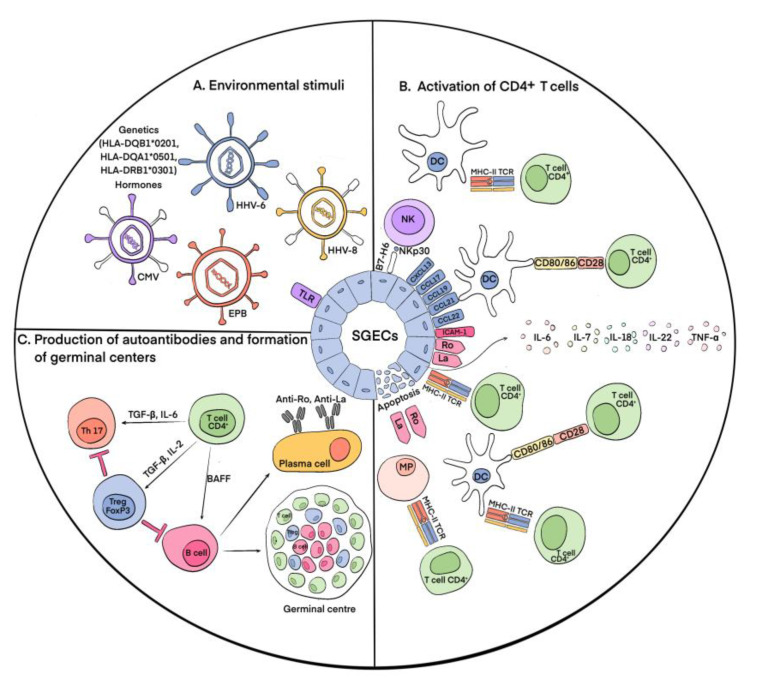
Main immunological events of pSS pathogenesis. (**A**) Environmental stimuli. According to present conceptions, environmental stimuli such as viral infections (CMV, EPB, HHV-6, and HHV-8), hormonal imbalance, genetic predisposition, and disturbances in the apoptotic system cause the activation of SGECs, resulting in the release of intracellular antigens Ro/La to the salivary cell surfaces. (**B**) Activation of CD4^+^ T cells. The appearance of Ro and La proteins on the outer surfaces of the membranes causes the autoactivation of cells of innate and adaptive immunity. At the same time, stimulus-activated signaling through TLR increases the expression of autoantigens Ro/La on the cell surfaces and causes apoptotic death of SGECs. As a result, apoptotic molecules and vesicles containing Ro/La are released from the cells. Autoactivation of CD4^+^ T lymphocytes occurs through MHC-II molecules, which are expressed on the surfaces of SGECs. SGECs also express chemokines CXCL13, CCL17, CCL19, CCL21, and CCL22, which along with the presence of autoantigens outside the cells leads to the accumulation of DCs in salivary glands, their interaction with CD4^+^ T cells, and their subsequent activation. In addition, SGECs express B7-H6, which contributes to the attraction of NK cells, their interaction with DCs, and the activation of CD4^+^ T cells. The autoantigens Ro/La outside of the SGECs lead to the recruitment of macrophages (MPs) and their interaction and activation of CD4^+^ T cells. ICAM1 expression by SGECs contributes to the concentration of lymphocytes in the focus of inflammation and the production of various proinflammatory cytokines: IL-6, IL-7, IL-18, and IL-22. (**C**) Production of autoantibodies and formation of germinal centers. Activated CD4^+^ T cells under the action of IL-6 and TGF-β are differentiated into Th17 cells, which, in turn, can be converted into Tregs under the influence of TGF-β and IL-2. With the participation of BAFF, CD4^+^ T cells activate B lymphocytes, which ultimately leads to the formation of autoantibodies and germinal centers.

The process of pSS development remains the subject of intensive study and research, especially for immunologists. The primary event in the development of pSS is the damage to and death of the epithelial tissue cells of the exocrine glands [19] as a result of the action of various external environmental stimuli (Figure 1). This process results in the autoactivation of immune cells, which can be indirectly measured via hypergammaglobulinemia, cryoglobulinemia, and the production of various autoantibodies in the peripheral blood and salivary glands of patients with pSS [20]. It is known that, for example, some latent viral infections (the cytomegalovirus (CMV), Epstein-Barr virus (EPB), and human herpesvirus types 6 and 8 (HHV-6,8)) cause the accumulation of viral genetic material in the salivary glands, which leads to the movement of antigens from the cytoplasm (Ro52 and Ro60) and from the nucleus (La and Ro60) to the cytoplasm and then to the cell surface, which has been shown primarily in salivary gland epithelial cells (SGECs) [21,22]. In addition to the viral hypothesis, which experimentally confirms the possibility of the release of intracellular Ro/La antigens to the cell surface and the appearance of Ro/La autoantibodies in the peripheral blood, there is also evidence that the apoptosis of SGECs results in the release of intracellular antigens to the cell surface, making them a target for autoantibody production [23]. The appearance of Ro/SSA and La/SSB proteins on the outer surface of the membrane causes the autoactivation of innate (dendritic cells (DCs), macrophages, and natural killer (NK) cells) and adaptive (T and B lymphocytes) immune cells via these proteins. In addition, SGECs express Toll-like receptors (TLRs), which increases their susceptibility to viral infections in pSS patients [24]. At the same time, the activation of Toll-like receptor 3 (TLR3) causes the apoptotic death of SGECs and the production of interferon 1 (IFN-1), which induces inflammatory processes, and B cell activating factor (BAFF), which promotes the differentiation of B lymphocytes [25,26]. The activation of signaling through TLR3 itself contributes to an increase in the expression of the autoantigens Ro/SSA and La/SSB in SGECs [27]. It also leads to the enhanced expression of molecules of the major histocompatibility complex II (MHC-II) and costimulatory molecules B2 and CD40 in affected SGECs [28]. The activation of TLR-2 via bacterial peptidoglycan, zymosan, and TLR-4 via lipopolysaccharides results in the expression of mediators of immune activation, such as intercellular adhesion molecule 1 (ICAM-1) [29]. The autoactivation of CD4^+^ T lymphocytes occurs through MHC-II molecules, and depending on the cytokine environment, they further differentiate into Tregs or T helper cells (Ths) [30]. In general, the role of the La/SSB antigen in the development of autoimmunity in pSS is confirmed by the hypomethylation of the promoter of this gene, which leads to its increased expression in salivary gland tissue [31] and indicates the involvement of genetic factors in the development of this pathology. The genetic prerequisites for enhanced SGEC death are also specific HLA alleles: HLA-DQB1*0201, HLA-DQA1*0501, and HLA-DRB1*0301 [32]. Studies have also identified variants of the IRF5, STAT4, EBF1, FAM167A-BLK, TNFSF4, CHRM3, and LAMP3 genes that increase the risk of developing this disease [33]. IRF5, IL12A, and STAT4 are involved in the IFN-1 signaling pathway. It was identified that the apoptosis of epithelial cells in pSS is also affected by sex hormones, which was confirmed by observations of lacrimal gland acini apoptosis during ovariectomy in mice [34].

As mentioned above, the destruction of exocrine gland cells triggers a cascade of immune reactions. Furthermore, activated and damaged SGECs express apoptotic molecules and release exosomes and apoptotic vesicles containing Ro/SSA and La/SSB autoantigens. SGECs express on their surfaces C-X-C motif chemokine type 13 (CXCL13), chemokine (C-C motif) ligand 17 (CCL17), chemokine (C-C motif) ligand 19 (CCL19), chemokine (C-C motif) ligand 21 (CCL21), and chemokine (C-C motif) ligand 22 (CCL22), which promote the recruitment of DCs and T cells to salivary glands. Accumulating there, DCs initiate an immune response [35]. Autoantigens activate plasmacytoid dendritic cells (pDCs), which cause the production of IFN-1, which supports inflammatory activity [36]. IFN-1 also induces the production of BAFF by circulating monocytes and DCs, which promotes the activation and differentiation of B cells into antibody-secreting plasma cells. Follicular DCs contribute to the survival and proliferation of B cells during the formation of ectopic lymphoid tissue [37]. Upon the activation of macrophages, inflammatory IL-1 and TNF-α are secreted, which leads to the destruction of glandular epithelial cells. Their number in pSS is directly correlated with the assessment of MSG lesions [38]. The number of NK cells is increased in the MSGs of patients [39]. They interact with DCs and SGECs, which leads to the subsequent activation of both innate and adaptive immunity [40]. Innate lymphoid cells contribute to the formation of ectopic lymphoid tissue [41]. Among the cells of adaptive immunity, various populations of B cells are involved in the pathogenesis of pSS; however, in the initial stages, T lymphocytes are dominant. CD4^+^ and CD8^+^ T lymphocytes are activated due to the presentation of autoantigens by DCs and macrophages. At the same time, CD4^+^ T cells are also activated directly by epithelial cells expressing MHC-II. SGECs also express intercellular adhesion molecule 1 (ICAM1), which contributes to the concentration of lymphocytes in the focus of inflammation, and produce various cytokines, including IL-6, IL-7, IL-18, and IL-22, which play an important role in the development of T- and B-cellular immune responses [42]. The rate of CD4^+^ T cells can reach more than 75% during infiltration to exocrine glands. The cells differentiate into T helper (Th) types 1 (Th1) and 2 (Th2), producing proinflammatory and anti-inflammatory cytokines [43,44]. CD4^+^ T cells also differentiate into Th17 cells, producing mediators such as IL-17, TNF-α, IL-22, and IL-26, contributing to the maintenance of the inflammatory process [45]. T-follicular helpers (Tfhs) promote the proliferation and differentiation of B cells in lymphoid infiltrates [46]. The activation of T and B cells results in the formation of ectopic germinal centers and the differentiation of plasma cells.

Thus, the main event of immunopathogenesis in the exocrine glands in pSS is the activation of epithelial cells and the subsequent development of the local immune response. As a result, a large number of autoantibodies produced by plasma cells bind to autoantigens released by damaged epithelial cells, increasing their damage and causing dysfunction. During this process, among CD4^+^ T cells, an important role belongs to regulatory T cells (Tregs). Despite the undeniable fact that Tregs are essential for the regulation of immunological self-tolerance and homeostasis [47], their actual contribution in pSS has yet to be specified.

## 3. Regulatory T Cells (Tregs)

The maintenance of self-tolerance is regulated via several processes, one of which is the suppression of proliferation and the activity of autoreactive T cells. Tregs play a leading role in this mechanism. The significance of Tregs for immune balance is constantly being supported by new data indicating that they are involved in almost all cases in which suppression of the immune response takes place, for example, in allergic processes, infections, antitumor immunity, and autoimmune diseases [48,49].

### 3.1. Differentiation and Activation of Tregs and the Role of FoxP3

As mentioned above, during the pathogenesis of pSS, CD4^+^ T lymphocytes are activated through MHC class II molecules expressed on the surfaces of macrophages and the epithelial cells of exocrine glands and through DCs. Furthermore, CD4^+^ T cells have several possible ways of differentiating into Th1 and Th2 cells, Th17 cells, Tfh cells, and Tregs [19]. The development of Tregs is associated with the actions of certain cytokines and costimulatory molecules [50].

**Figure 2:**
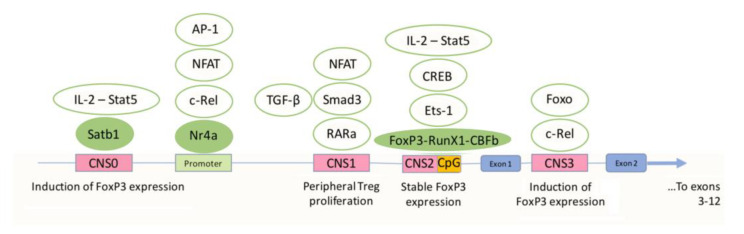
Regulatory sequences of FoxP3. The key regulatory regions of the FoxP3 gene include several conserved noncoding DNA sequences (CNS 0–3). CNS 0–1 and 3 contribute to the induction of FoxP3 expression, wherein CNS1 promotes the proliferation of peripheral Tregs, and CNS2 determines the maintenance of a high and stable level of FoxP3 expression. CpG island as a part of CNS2 is responsible for methylation. A high degree of CpG demethylation is associated with elevated expression of FoxP3. Direct activators of the CNS are shown as green ellipses, while indirect activators are shown as white ellipses.

Treg differentiation has been reported to require the stimulation of the T-cell receptor (TCR) via a small number of high-affinity ligands [51]. By receiving TCR signals, T cells become activated. Activated cells produce CD25, thereby becoming Treg precursor cells. Further stimulation of the TCR initiates the transcription of forkhead box P3 (FoxP3), a transcription factor that plays a master role in the differentiation of Tregs, determining their size and stability as well as the acquisition of their suppressive function [52]. Studies have shown that FoxP3 is able to bind to a variety of genomic sites and interact with various binding partners to achieve the proper differentiation and acquisition of functional Treg activity. By interacting with transcriptional coactivators and corepressors, including those induced by the TCR (for example, NF-κB (c-Rel), nuclear factor of activated T cells (NFAT) and AP-1), FoxP3 helps to increase or suppress gene expression, while interaction with chromatin remodelers promotes the availability of genes for their interaction with other transcription factors [53,54,55]. When FoxP3 transcription is stabilized, its regulation occurs through so-called autoregulatory transcription factors that bind to key regulatory regions of the FoxP3 gene. The genomic region of the Foxp3 locus has several conserved noncoding sequences (CNS 0–3) (Figure 2).

CNS 1 and 2 are located in the region of intron 1, and CNS3 is located in the intron after the first exon. CNS 0–1 and 3 contribute to the induction of FoxP3 expression, while CNS2 determines the maintenance of a high and stable level of FoxP3 expression [56]. c-Rel binds to the promoter of FoxP3 and CNS3, which promotes the differentiation of Treg precursors and the activation, survival, division, and functional activity of Tregs [57]. In addition to c-Rel, NFAT, activator protein-1 (AP-1) and nuclear receptor 4A (Nr4a) also bind to the promoter. It has been suggested that Nr4a regulates TCR-induced FoxP3 expression. Special AT-rich sequence-binding protein-1 (Satb1) binds to CNS0 and promotes FoxP3 expression in the thymus. In addition, Satb1 causes the interaction of other transcription factors with this region [58]. FoxP3 expression also depends on the interaction of CNS0 with Interleukin-2 (IL-2) and signal transducer and activator of transcription 5 (Stat-5). In CNS0, IL-2 and Stat-5 promote FoxP3 expression during Treg differentiation, while in CNS2, on the contrary, this is carried out in already mature, differentiated cells [59]. CNS2 also binds Foxp3, Runx1- Core binding factor β (CBFβ), and Ets-1. Runx1 activates FoxP3 transcription. In addition, the conserved CpG island in the CNS2 region was found to be hypomethylated in Tregs and hypermethylated in conventional (nonregulatory) CD4^+^ T cells. The methylation status of the highly conserved region, also called the Treg-specific demethylated region, determines the level of FoxP3 expression [60]. NFAT, mothers against decapentaplegic homolog 3 (Smad3), and retinoic acid receptor-alpha (RARa) bind to CNS1. Smad3 is activated by TGF-β. The interaction of retinoic acid with RARa increases Smad3 expression, which in turn leads to an increase in Foxp3 expression [61]. In summary, TCR-induced transcription factors integrate the TCR and costimulatory signaling pathways through CNS0–3 to initiate Foxp3 transcription. However, once Foxp3 transcription becomes stable over time, an autoregulatory feedback system is established that maintains transcription through epigenetic modifications of the Treg-specific demethylated region in CNS2 [62].

The cytokines transforming growth factor-beta 1 (TGF-β1) and IL-2 are also necessary for the development of Tregs. TGF-β1 induces NFAT and Smad3 binding to CNS1 [63]. IL-2 limits the differentiation of activated CD4^+^ T cells into Th17 cells, subsequently maintaining the stability and proliferation of Tregs. In addition, retinoic acid produced by DCs, like IL-2, prevents the differentiation of CD4^+^ T lymphocytes into Th17 [64] and, accordingly, in combination with TGF-β1 and IL-2, ensures the formation of Tregs. Because the developmental pathways of Th17 and Tregs are connected, an increased number of pathogenic Th17 cells producing IL-17 is often accompanied by a reduced number of Tregs: TGF-β is required for the differentiation of both Th17 and Tregs, but the presence of IL-6 leads to the formation of Th17 cells, and its absence leads to the formation of Tregs [65]. Obviously, the balance between the two populations of cells can be easily disturbed, leading to the prevalence of pathogenic cells and, consequently, the development of autoimmunity. Moreover, it is well known that FoxP3^+^ Tregs can be converted into inflammatory IFN-γ FoxP3^+^ Tregs or IL-17^+^ FoxP3^+^ Tregs via IL-12 stimulation or in the absence of TGF-β. Expression of IFN-γ does not inhibit the ability of Tregs to suppress T-cell proliferation but reduces the number of Tregs and their level of FoxP3 expression. IL-12 suppresses Treg proliferation and reduces the production of IL-2 by CD8^+^ cells, whose function is to suppress Th17 differentiation. IL-6, together with IL-1, induces the genetic reprogramming of cells into FoxP3^+^ Tregs [65].

Thus, increased secretion of TGF-β1 in pSS, along with the presence of IL-2, FoxP3 expression, and retinoic acid, determines the differentiation of CD4^+^ T lymphocytes into Tregs. Furthermore, Tregs start implementing their functions: the suppression of proliferation and functioning of effector T lymphocytes.

### 3.2. Mechanisms of Suppressive Action of Tregs

**Figure 3:**
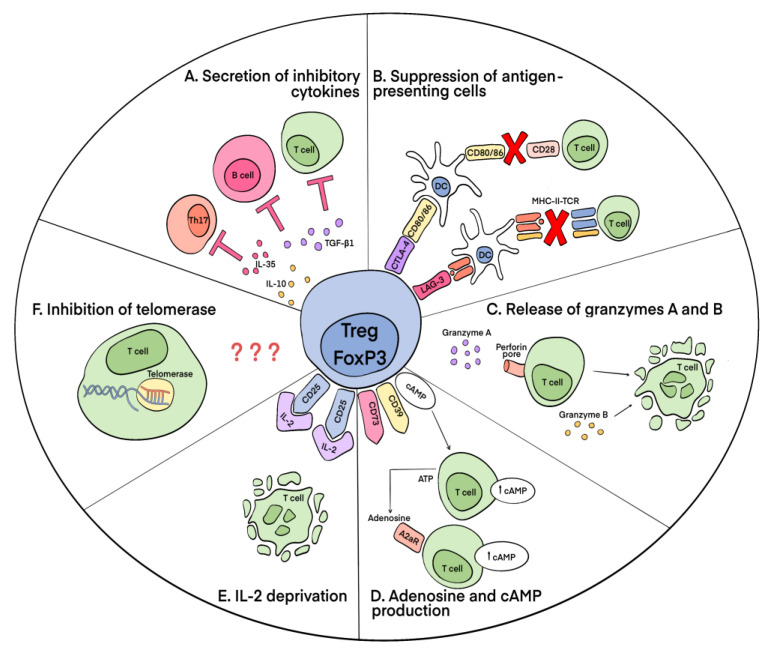
Mechanisms of suppressive activity of Tregs on effector lymphocytes. (**A**) Secretion of inhibitory cytokines. Tregs produce IL-10, IL-35, and TGF-β1. TGF-β1 inhibits the growth and differentiation of T and B lymphocytes. IL-10 and IL-35 suppress Th17. (**B**) Suppression of antigen-presenting cells. Suppression via cell contact is achieved via the expression of CTLA-4 and LAG-3 on the Treg membrane. CTLA-4 binds to costimulatory CD80/86 molecules on DC surfaces, which leads to the inability of CD80/86 on DCs to interact with CD28 on effector T cells and to activate them. LAG-3, having high homology to the CD4 structure, binds with high affinity to MHC-II molecules of DCs and makes it unavailable to the TCRs of effector lymphocytes. (**C**) Release of granzymes A and B. Tregs secrete serine proteases GrA and GrB, which induce apoptosis of effector T cells in a perforin-dependent (GrA) and perforin-independent (GrB) pathways. (**D**) Adenosine and cAMP production. Treg-produced exoenzymes ectoapyrase CD39 and ecto-5’-nucleotidase CD73 form adenosine, which binds to the adenosine receptor A2 on the surfaces of effector T lymphocytes. Activation of the A2 receptor leads to an increase in the intracellular concentration of cAMP and results in impaired cell proliferation. In addition, Tregs contain a large amount of cAMP, which disrupts the function of effector cells in a contact-dependent manner. (**E**) IL-2 deprivation. Tregs consume large amounts of IL-2 (due to the presence of high CD25), which leads to IL-2 deprivation and the death of effector cells. (**F**) Inhibition of telomerase. Tregs activate EndoG endonuclease, which induces alternative splicing of the catalytic subunit of telomerase. This leads to cell cycle arrest and the transition of cells to replicative senescence and apoptotic death.

Several mechanisms of the Treg-mediated suppression of effector autoreactive lymphocytes are known. They can be divided according to the main macromolecules involved in their activity: the secretion of inhibitory cytokines and granzymes, the implementation of suppressive activity through intercellular contact, the disruption of the metabolism of effector T lymphocytes, and the inhibition of telomerase (Figure 3).

Suppression through cell contact is achieved via the expression of Tregs, such as the molecules cytotoxic T-lymphocyte-associated antigen 4 (CTLA-4) [66] and lymphocyte activation gene-3 (LAG-3 (CD223). CTLA-4 binds to costimulatory B7 molecules on the surfaces of antigen-presenting cells (APCs), DCs, and monocytes, preventing their interaction with CD28 on T cells and thereby blocking their activation. Without appropriate costimulation, T cells are tolerant toward the antigen presented to them. The expression of CTLA-4 is increased in patients with pSS [67]. LAG-3, having high homology to the CD4 structure, binds with a high affinity to the MHC-II molecules of APCs, thereby triggering an inhibitory signaling pathway that prevents the activation of APCs and, accordingly, T lymphocytes [68]. The expression of LAG-3 by Tregs is very high [69].

As for metabolic disruptions, the exoenzymes ectoapyrase CD39 and ecto-5’-nucleotidase CD73, produced by Tregs, form adenosine, which binds to the adenosine receptor A2 on the surfaces of effector T lymphocytes, increasing the intracellular concentration of cAMP in them and thereby leading to impaired proliferation [70]. In addition, Tregs contain a large amount of cAMP. Accordingly, when they interact through gap junctions with effector T cells, the functioning of the latter is also impaired [71]. Additionally, increased expression of CD25 by Tregs induces T-lymphocyte cytokine-deficient apoptosis. This is explained by the high consumption by Tregs of IL-2, which subsequently leads to the impaired proliferation and death of effector cytokine-producing CD4^+^ T cells [72].

Suppression via the secretion of inhibitory cytokines is performed via the production of IL-10, IL-35, and TGF-β1 by Tregs. IL-35 and IL-10 contribute to the suppression of Th17 [73,74]. TGF-β1 inhibits the growth and differentiation of T and B lymphocytes [75]. In addition, Tregs secrete serine proteases granzyme A (GrA) and granzyme B (GrB), which cause the apoptosis of effector T cells via perforin-dependent (GrA) and perforin-independent (GrB) pathways [76].

The discovery by researchers of the ability of Tregs to inhibit telomerase in T and B lymphocytes and NK cells deserves special consideration. It has been shown that telomerase activity in target cells decreases as a result of the alternative splicing of its catalytic subunit human telomerase reverse transcriptase (hTERT) induced by the Treg-mediated activation of the apoptotic endonuclease EndoG. The suppression of telomerase is a contact-independent mechanism of Treg suppressive activity. The inhibition of telomerase leads to a decrease in the length of telomeres in cells, cell cycle arrest, and the transition of cells to replicative senescence and apoptotic death [77,78]. The exact mechanism that allows Tregs to activate EndoG in target cells and mediator molecules currently remains to be determined.

### 3.3. The Role of FoxP3 Protein in the Regulation of the Functional Activity of Tregs

The FoxP3 protein, being a key regulator of Treg differentiation, as well as their most specific marker, is necessary for Tregs to acquire their functional activity. It is noteworthy that for the implementation by Tregs of their suppression function, the amount of expressed FoxP3 becomes critical. This was proven via a study on the failure of Tregs’ suppression of spontaneous autoimmunity with an approximately 10-fold decrease in FoxP3 protein expression due to a change in the 3’ untranslated region (UTR) of the FoxP3 gene [79]. FoxP3 enhances and stabilizes the expression of Treg genes encoding cell surface markers and cell-secreted molecules such as Fgl2 (fibrinogen-like protein 2), CD73, CD39, and CTLA4, which are direct participants in the mechanisms of Treg-mediated suppression. At the same time, FoxP3 enhances the repression of TCR-activation-dependent proinflammatory cytokines, including IL-4, IFN-γ, TNF-α, IL-17, and IL-21. In addition, by modulating the cell surface and signaling molecules, Foxp3 alters the Treg response to external stimuli to help maintain normal homeostasis and Treg stability. A significant contribution to adaptation to external stimuli is made by the FoxP3-induced repression of cyclic nucleotide phosphodiesterase 3B (PDE3B), which leads to the conservation of a large amount of cAMP in cells [80].

An object of study for researchers is the alternative splicing of FoxP3 mRNA, which has a great impact on Treg biology. It was shown that in human cells, FoxP3 is expressed mainly in two isoforms, full-length (full-length–FOXP3FL) and with a deletion of exon 3–FOXP3Δ3, which are the results of the alternative splicing of FoxP3 mRNA. The previously described isoforms FOXP3Δ3Δ8 and FOXP3Δ8, according to the available data, do not play a role in determining the functional activity of Tregs [81]. However, the expression profiles of FoxP3 splice variants change upon ex vivo Treg maturation from CD4^+^ T cells [82]. It should be clarified that in the literature, there are differences in the numbering of these exons, and exons 3 and 8 in some sources are defined as 2 and 7, respectively. It has been reported that the coexpression of the FOXP3FL and FOXP3Δ3 isoforms becomes crucial for Tregs to acquire their phenotype and implement their suppressive activity [83]. In this case, the production of proinflammatory cytokines such as IL-17 and IL-22 is reduced. In addition, the FOXP3Δ3 isoform induces the transcription of glycoprotein A repetitions predominant (GARP), one of the key molecules involved in the suppressive mechanisms of Tregs [84]. At the same time, GARP in the presence of IL-2 promotes Treg differentiation and inhibits Th17 differentiation. In addition, the lack of a binding site in FOXP3Δ3 for the transcription factor receptor-related orphan nuclear receptor (RORγT) leads to increased secretion of IL-17, which suggests a significant role of the regulated expression of FOXP3Δ3 in establishing a balance between Treg and Th17 cells [85]. Thus, the modulation of alternative splicing, leading to an increase in the expression of and being important for Treg differentiation and functioning of FoxP3 isoforms, can become a promising approach in the treatment of diseases with an impaired T-cell component of immunity.

## 4. Involvement of Tregs in pSS

Tregs are the main cells involved in the regulation of loss of immunological tolerance in pSS. With the progression of the disease, the tissue of the exocrine glands is destroyed, which is associated with lymphocytic infiltrates, consisting mainly of activated T and B lymphocytes. In the early stages of the disease, autoreactive T cells predominate, in the suppression of which Tregs play an indispensable role.

### 4.1. Heterogeneity of Tregs in pSS

**Figure 4:**
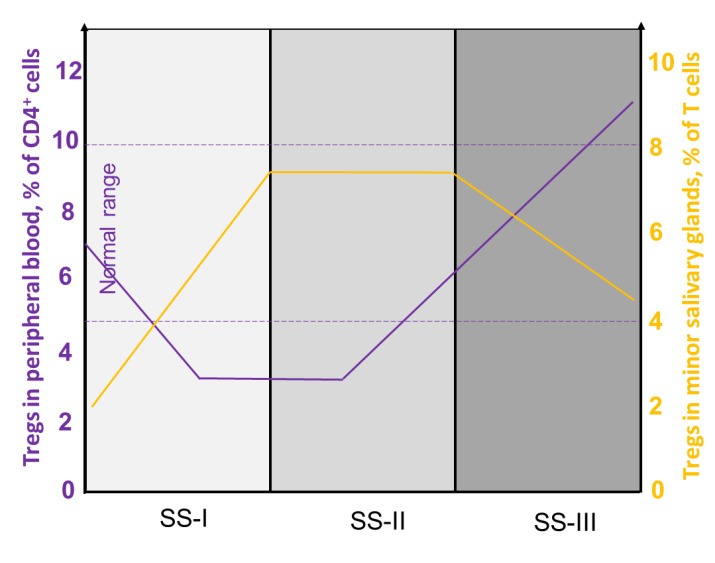
Proportion of Tregs in peripheral blood and in minor salivary glands depending on the stage of the disease. The numbers of Tregs in the PB of patients depend on the stage of the disease. The lowest mean percentage of Tregs to CD4^+^ cells (3.89–4.29) can be observed in the group with intermediate MSG lesions (SS-II), whereas the highest (8.1–9.84) is in patients with advanced MSG lesions (SS-III). In patients with a mild degree of damage, the mean percentage of Tregs to CD4^+^ cells is 1.66–2.72. The largest number (6.45–8.11) of Tregs as a percentage of CD3^+^ T cells can be found in MSG tissues with an intermediate degree of damage (SS-II), and the smallest (1.66–2.72) is found in mildly affected tissues (SS-I). FoxP3^+^ cell infiltration of MSG tissues with an advanced degree of damage (SS-III) is significantly reduced (3.61–5.29) compared with SS-II.

**Figure 5:**
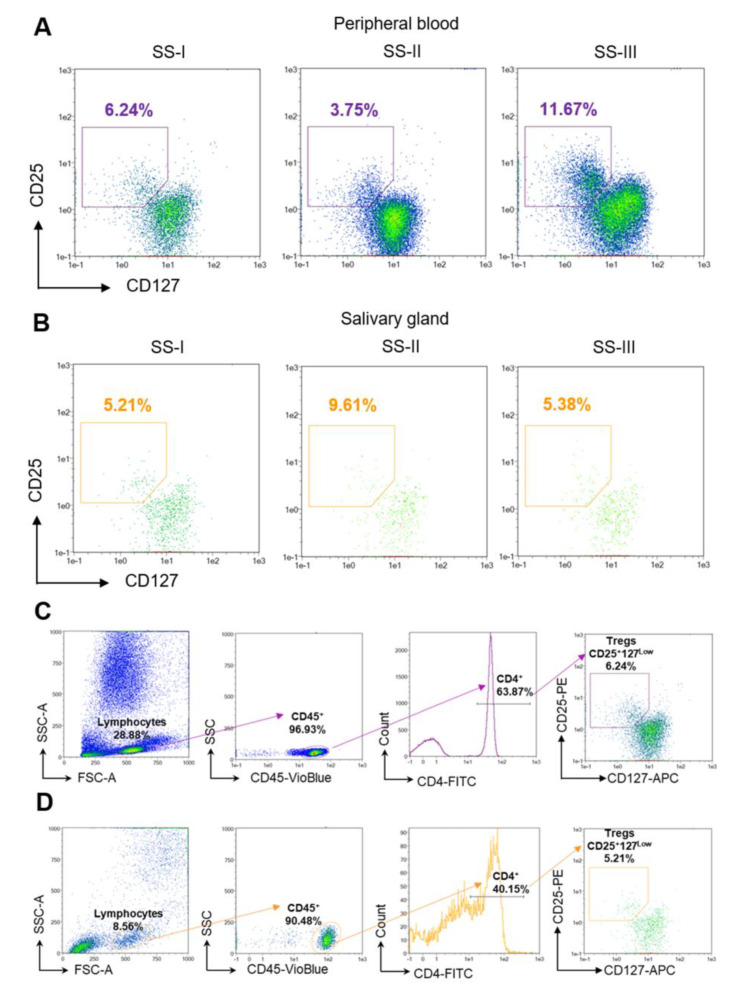
Flow cytometry plots of CD25^+^CD127^low^ Tregs demonstrating the changes in the proportion of Tregs in (**A**) peripheral blood and in (**B**) minor salivary glands, depending on the stage of the disease. Gating strategy for Treg determination in (**C**) peripheral blood and (**D**) biopsy of salivary glands from patient with pSS. One representative example from a single patient is shown. The data were provided by Medical Center Ltd.

The number and evaluation of the suppressive activity of Tregs in patients with pSS were studied in the cells of MSGs and the peripheral blood (PB). The number of CD4^+^CD25^+^FoxP3^+^ Tregs in MSGs is increased in patients with pSS, whereas infiltration rates depend on the degree of glandular damage. The largest number of FoxP3^+^ cells was found in MSG tissues with an intermediate degree of damage (SS-II), and the smallest was found in mildly affected tissues (SS-I). FoxP3^+^ cell infiltration of MSG tissues with a severe degree of damage (SS-III) was significantly reduced compared with SS-II, which may be due to the involvement of Tregs in more complex processes of immune deregulation [15]. Additionally, a lower number of CD4^+^CD25^+^FoxP3^+^ Tregs correlates with the presence of factors predicting the development of lymphoma, primarily with an increase in the salivary glands [15]. As for the number of CD4^+^CD25^+^FoxP3^+^ Tregs in the PB, the results are controversial. Several studies [86,87,88,89,90] revealed a reduction in CD4^+^CD25^+^FoxP3^+^ Tregs in the PB upon disease progression, whereas other results [91,92] demonstrated an increase in the numbers of these cells. Such discrepancies may be explained by different groups of patients involved in these studies. With a reduced number of Tregs in the PB, their content in MSGs in SS-II is increased, which may indicate that Tregs leave the circulation and accumulate in inflamed tissue (Figure 4 and Figure 5) or can develop/maturate onsite from CD4^+^ precursors. In patients with a high degree of MSG damage (associated with SS-III), a large number of FoxP3^+^ cells are found in the PB, which may be due to systemic lesions and massive Treg maturation [15].

In some patients with pSS, disturbances occur not only in the activity of the exocrine glands but also in the function of the digestive, musculoskeletal, respiratory, urinary, endocrine, peripheral, and central nervous systems. At the same time, they have corresponding differences in the number of Tregs. The content of CD4^+^CD25^+^FoxP3^+^ Tregs in the PB in patients with extraglandular manifestations (EGMs) was significantly higher than that in patients without such manifestations. At the same time, the total numbers of CD4^+^CD25^+^FoxP3^+^ cells were reduced in both subgroups of patients. The suppressive activity of CD4^+^CD25^+^FoxP3^+^ Tregs in patients with both extraglandular manifestations and their absence was lower than that in the control group [86].

Among cells with the Treg phenotype and a low level of CD25 expression, T lymphocytes expressing the glucocorticoid-induced TNF-receptor-related (GITR) protein, CD4^+^CD25^low^GITR^+^, were found. GITR is a surface molecule that can identify T cells with regulatory activity. These cells express FoxP3, TGF-β, and IL-10, which are key cytokines involved in the mechanisms of suppression, and they are functionally active and able to inhibit the proliferation of effector T lymphocytes. Their number is increased in the MSGs and PB of patients with low disease activity (ESSDAI ≤ 2) [90]. Another marker of activated Tregs is the Helios protein, and it has been proven that Helios^+^FoxP3^+^ Tregs have more pronounced suppressive activity than Helios^-^FoxP3^+^ Tregs. This may be due to the fact that Helios binds to the FoxP3 promoter, enhancing its expression [93]. Peripheral blood levels of Helios^+^FoxP3^+^ Tregs are increased in patients with pSS. Their number is inversely proportional to the disease activity according to the ESSDAI [94]. The number of type-1 Tregs (Tr1) is significantly reduced in the blood of patients with pSS. Their differentiation is induced by IL-27, characterized by a high level of IL-10 expression and the ability to inhibit T-cell responses. At the same time, ex vivo Tr1 polarization is inhibited by proinflammatory IL-12. Anti-IL-12 therapy in mice has been shown to significantly increase Tr1 cells and improve salivation, as well as reduce inflammation and damage to the salivary glands, which may also be of interest in the search for new treatment strategies targeting T cells [95]. The difficulty in the comparison of the data between different studies relies on the use of different cell markers for measuring Treg numbers in the blood. The Tregs numbers and suppressive activity in patients with pSS are presented in the Table 1. They are measured in comparison with healthy donors.

### 4.2. Tregs and Ectopic Germinal Centers

Lymphoid infiltrates in pSS are organized into structures similar to the germinal centers of peripheral lymphoid tissue. They arise after antigenic stimulation and provide the environment necessary for B-cell proliferation, somatic hypermutagenetic processes, and antibody class switching, thereby leading to disease progression. Recent studies have shown that approximately 25.1 ± 5.0% of patients with pSS have ectopic germinal centers (GCs) in their salivary glands. Lymphoplasmacytic infiltration foci in patients with GCs were 1.25 units higher than in patients without GCs, and they also have an increased risk of developing lymphoma [96,97]. 

**Figure 6:**
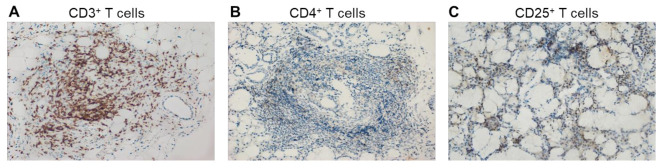
Immunohistochemical staining of paraffin-embedded MSG biopsies that were obtained from patients with pSS. Microscopic and immunohistochemical profile of focal sialadenitis and confluent lymphocytic infiltrate with germinal centers of the minor salivary gland. Positive reaction with (**A**) mAbs against CD3; (**B**) mAbs against CD4; and (**C**) mAbs against CD25. Large positive area, X200. The data were provided by Medical Center Ltd.

GCs are unique structures formed by antigen-specific B and T cells, which are previously activated in the early stages of the immune response. They consist mainly of T and B lymphocytes and the CD3^+^ T cells/CD20^+^ B cells ratio is negatively associated with the stage of the disease. Among CD3^+^ T cells, CD4^+^ T cells are dominant. The numbers of total CD3^+^ T cells and the subpopulation of CD4^+^ T cells in infiltrates decrease with the progression of pSS. The incidence of CD25^+^ Tregs reaches its highest value in intermediate (SS-II) lesions (Figure 6). The numbers of macrophages in lymphoplasmacytic infiltrates change in accordance with the progression of the disease, whereas the numbers of CD8^+^ T cells, fDCs, and NKs remain unchanged [38]. Among the cells of ectopic GCs, follicular T regulatory cells (Tfr) deserve special attention, the number of which is directly dependent on the presence of GCs [98]. Tfr cells are defined as an effector subset of Tregs that express C-X-C chemokine receptor type 5 (CXCR5), which directs them to migrate to GCs. They originate from Foxp3^+^ natural Treg precursors and are phenotypically characterized by the expression of CXCR5, programmed cell death 1 (PD-1), inducible T-cell costimulator (ICOS), and the transcription factor B-cell lymphoma 6 (Bcl-6) [99,100]. The function of Tfr cells is to regulate the differentiation of follicular T helper cells (Tfhs). Tfh cells in GCs interact with B cells, producing a large amount of IL-21, thereby supporting B-cell proliferation and plasma cell differentiation [101]. In patients with pSS, the number of Tfh cells in the blood and glandular tissues is increased [102]. Tfr cells suppress the differentiation and proliferation of Tfh cells and limit the number of B cells, thereby controlling B-cell selection in GCs and limiting the proliferation of antigen-nonspecific clones. The PB and MSGs of patients with pSS are rich in Tfr cells. Patients had a significantly increased Tfr/Tfh ratio compared with the control group in [103]. In human lymph nodes, Tfr cells are usually located on the border between the T-zone and the B-zone of the follicle [104]. Due to their location, Tfr cells control the activity of GCs by interacting with B cells and migrating Tfh cells into or outside of GCs. Tfr cells, like Tfh cells, mainly realize their activity within the lymphoid tissue; however, a small amount of circulating Tfr (cTfr) and Tfh (cTfh) cells in the blood has been found. cTfr can, for a long time, remain in the blood and then be recruited into GCs [105]. In addition, they have a weaker suppressive capability than tissue Tfr cells, expressing lower levels of ICOS than lymph node Tfr cells [106]. This can be explained by the observations that cTfr cells leave the lymphoid tissue as immature cells before B-cell interaction on the T-B border and full further differentiation into Tfr cells and the acquisition of the complete suppressive function [105].

## 5. Immunotherapy of pSS

Due to the observation that B cells play a crucial role in the development of ectopic lymphoid tissue and the overproduction of autoantibodies, therapy aimed at them is currently the most common. However, the mechanism of this therapy is based on the total elimination of B cells, which leads to a significant weakening of the immune system. In the initial stages of the disease, T lymphocytes, which mainly consist of CD4^+^ T cells, are the predominant cells in infiltrates, and in the subsequent stages, these activated T cells, therefore, activate B cells. As a result, researchers believe that treatment that targets, above all, T cells and then B cells or a combination of T- and B-cell therapies may be the most effective [107]. The tables below (Table 2 and Table 3) review the data on the mechanism and efficacy of pharmaceuticals used in the treatment of pSS or that are at the stage of clinical trials.

### 5.1. Pharmaceutical B Cell Therapy

The role of B cells in the pathogenesis of pSS makes them the primary target for therapy. Aberrant activation of B lymphocytes leads to the production of autoantibodies and changes in other serological characteristics (for example, hypergammaglobulinemia), the formation of GCs, and the development of extraglandular manifestations of the disease. In addition, some patients develop malignant MALT lymphomas due to B-cell hyperactivation. Currently, the mechanisms underlying B-cell therapy are B-lymphocyte depletion (Rituximab), the modulation of BCR (B-cell receptor) signaling (Epratuzumab), and the inhibition of BAFF (belimumab and ianalumab) (Table 2).

Therapy with rituximab helps to reduce the activity of B lymphocytes, reduce lymphocytic infiltrates and GCs, eliminate the symptoms of dryness, fatigue, extraglandular manifestations, and, accordingly, reduce the activity of pSS on the ESSDAI scale. However, conclusions about the effectiveness of this drug vary among researchers due to the use of different criteria for evaluating its effectiveness. Epratuzumab improves the function of the salivary and lacrimal glands, eliminates fatigue, and reduces the level of B cells without affecting T cells and immunoglobulin levels. Belimumab reduces parotid edema and B-cell activation biomarkers, with no improvement in salivary and lacrimal gland function. With the use of ianalumab, an antibody with a dual mode of action (BAFF receptor blockade and direct lysis of B cells via antibody-dependent cellular cytotoxicity), the activity of the disease according to the ESSDAI scale decreases, while there are no clear data on improvement in the function of the salivary and lacrimal glands. In the safety and efficacy results from a phase-2 proof-of-concept trial inhibitor of Bruton’s tyrosine kinase (BTK), remibrutinib showed improvement in disease activity according to the ESSDAI, in salivary flow, and in pathologically elevated immunoglobulins. Baminercept has a significant effect on the number of circulating B and T cells; however, there is no regression of extraglandular manifestations and improvement in the activity of the salivary and lacrimal glands.

### 5.2. T Cell Therapy

The search for T-cell therapy strategies has attracted the attention of researchers due to several significant events in the development and progression of pSS. First, in the early stages of the disease, the lymphoplasmacytic infiltrates in the exocrine glands consist predominantly of CD4^+^ T cells. Second, the hyperactivation of B cells, the significance of which was mentioned above, occurs as a result of the interaction between B cells and activated T cells. Based on this, the search for new targets for T-cell therapy appears reasonable and promising. 

#### 5.2.1. Pharmaceutical Drug Therapy

Existing drugs are mainly aimed at inhibiting T-cell proliferation by blocking the interaction of T lymphocytes with APC (abatacept, CFZ533) as well as with leukocyte function-associated antigen-3 (LFA-3) (Alefacept) (Table 2). However, CFZ533 also blocks T–B cell interaction, considering that CD40, the target of CFZ533, is also present in B cells. 

Studies have shown that the use of abatacept is most effective in the early stages of the disease. At the same time, there was a decrease in inflammation in the salivary glands, an improvement in salivation, and a decrease in the number of Tfh and Tregs, but no changes are observed in the foci of lymphoplasmacytic infiltration. Alefacept induces the depletion of CD4^+^ and CD8^+^ cells in psoriasis, and promising results are expected in pSS. CFZ533 (iscalimab) is in phase 2 of clinical trials. The results of the previous phase evaluated the safety, tolerability, and preliminary therapeutic efficacy of CFZ533 in patients with pSS. AMG557, a human monoclonal antibody, which targeted ICOSL on APC and B-cell surfaces, thereby blocking ICOS:ICOSL interaction, failed phase 2a of clinical trials [128].

The analysis of current data makes it possible to state that pSS is currently pathogenetically poorly understood and that the treatment of this disease is mostly symptomatic. This forces scientists to search for more promising and effective treatment strategies. Considering the undeniable contribution of the hyperproduction of autoantibodies to the progression of pSS caused by the excessive activation of T and B lymphocytes, it is widely accepted that a combination of therapies aimed at the T- and B-cell components of adaptive immunity may be the most effective. This seems especially reasonable given that lymphocytic infiltrates, which form during the development of pSS and cause dysfunction of the exocrine glands, in the early stages are mainly represented by CD4^+^ T lymphocytes. At the same time, these autoreactive T lymphocytes are suppressed by Tregs, which makes them an attractive target for therapy in the early stages of the disease. Approaches for this therapy are being actively developed for the treatment of autoimmunity and in transplantology, wherein Treg-mediated suppression and limiting of the immune response may be crucial. 

#### 5.2.2. Treg-Based Therapy

Several approaches for the induction of Treg expansion are known. IL-2-based approaches, as well as polyclonal, antigen-specific, or cell-engineered (CAR and TCR-transduced) cell-based approaches, are being tested in the fields of transplantation and for various autoimmune diseases [129]. Promising results have been shown in an open-label multicenter phase-I/IIa clinical trial using ovalbumin-specific Tregs (ova-Tregs) in Crohn’s disease (CD). Ova-Tregs cells were isolated from patients’ peripheral blood mononuclear cells (PBMCs), exposed to ovalbumin, and administrated intravenously. Administration of ova-Tregs to patients with refractory CD (CATS1) was well tolerated and demonstrated dose-related efficacy [130]. Along with that, it was reported that a highly specific RARa agonist induces the expression of integrin α4β7c on the Treg surface. Adoptive transfer of RARα-agonist-treated Tregs led to improved Treg trafficking to gut tissue in a humanized mouse model of colitis [131]. A clinical trial with the use of a RARα-agonist-treated polyclonally expanded autologous Treg product in CD is still ongoing [132]. The results of two clinical trials exploring the efficacy of Treg-based therapy in patients with type 1 diabetes (T1D) demonstrated safety [133,134]. Children treated with autologous ex vivo-expanded Tregs resulted in a lower requirement for exogenous insulin, with two children becoming completely insulin-independent after one year. It was also stated that repetitive administration of Tregs can prolong the survival of β cells in T1D [133]. Another study (phase I) with a combination of polyclonal Tregs and low-dose IL-2 for the treatment of T1D was completed, indicating the role of IL-2 in the expansion of exogenously administered Tregs and, at the same time, of cytotoxic cells [135]. Clinical trials in the phase-I/II stage (NCT02932826) with Tregs derived from the umbilical cord at high and low doses in a single infusion are ongoing. One case study demonstrated the effects of a single dose of ex vivo-expanded autologous Tregs in a patient with systemic lupus erythematosus (SLE) with active skin disease [136]. The study revealed the transient presence of infused Tregs in the peripheral blood, accompanied by increased percentages of highly activated Tregs in the diseased skin. Treg-cell accumulation in the skin was associated with a marked attenuation of the IFN-γ pathway and a reciprocal augmentation of the IL-17 pathway. However, the number of circulating labeled cells dropped at 4 weeks post-injection with a simultaneous marked increase in Tregs and IL-17 production in the skin biopsy. Some authors state that further studies on adoptive Treg therapy in SLE should be conducted to interpret these findings, avoiding existing limitations [136]. In a mouse model of collagen-induced arthritis, the adoptive transfer of antigen-specific Tregs reversed the disease’s progression by suppressing TNF-α. The results of this study confirm that antigen-specific Tregs might be a potential novel approach for the treatment of rheumatoid arthritis [137]. Trials using ex vivo-grown Tregs have proven the high suppressive activity and viability of the cells in multiple sclerosis [138]. However, many issues remain to be resolved. It is unclear which cell expansion strategy is optimal in each disease and which number and frequency of Treg injections are required for efficient tolerance induction. Moreover, some other limitations associated with the stability of Tregs in peripheral blood and their purity and potency as a cell product should be explored [139]. Regardless of the fact that, currently, there is no evidence of studying the use of Treg-based therapy in pSS, the successful and promising application of these cells in adoptive therapies for other autoimmune diseases makes it possible to consider this approach for pSS.

In addition, cytokines (mainly TNF, IFN, IL-1, IL-2, IL-6/12, IL-10, and IL-17) have also been studied as possible therapeutic targets for pSS [140]. It was suggested that their inactivation could lead to an improvement in the clinical symptoms of xerostomia and xerophthalmia; however, anti-TNF therapy did not prove to have efficacy in clinical trials [141,142]. Genetically engineered drugs are being developed to help avoid systemic immune suppression and severe side effects. In attempts to deliver adenoviral vectors expressing the IL17R:Fc fusion protein and encoding IL-27, which are the demonstrated inhibitors of Th17 activity, the mice that received these vectors showed a decrease in disease progression. In addition, an AQP1 (aquaporin-1)-encoding adenoviral vector was used to improve the function of salivary and lacrimal glands. These approaches have shown great potential for further development as a gene therapy for pSS [107].

We hope that the more detailed information on the role of Tregs in pSS that we provided in this review can help develop new approaches to the treatment of pSS, including the possibility of using adoptive cell therapy using Tregs, and improve the effectiveness of the treatment.

## 6. Future Perspectives on Treg Studies in pSS

Studying the reasons that lead to the failure of the suppressive activity of Tregs against T lymphocytes in GCs is interesting for further research. It is possible that Tregs in GCs are not as functional as those in the PB. This issue has yet to be explored. Since FoxP3 is a key regulator of Tregs, it is necessary to determine its contribution to their biology in GCs. Recent studies on FoxP3 splice variants suggest a significant role of the splice isoforms of this transcription factor in the functioning of Tregs in pSS, including GCs. Thus, such an approach of modulating the alternative splicing of exons in FoxP3 mRNA by splice-switching oligonucleotides or gene editing via CRISPR/CAS9 may become a promising strategy for producing Tregs with increased suppressive activity for regenerative Treg-cell therapy. The investigation of different strategies for ex vivo Treg expansion should be continued in order to develop diverse approaches to increase the number and suppressive activity of various Treg subpopulations. Beyond that, future studies on the possible application of Tregs with a chimeric antigen receptor (CAR) in therapy for pSS are of particular interest. The further development of engineered CAR Tregs may contribute to increasing Tregs’ suppressive function in pSS. Machine learning techniques could facilitate the development of treatment strategies for patients with pSS by being implicated in risk prediction, the monitoring of disease progression and outcomes, and management.

## Figures and Tables

**Table 1:** The changes in Tregs number and suppressive activity in patients with pSS. The comparison is carried out relative to healthy donors.

Treg Population in Peripheral Blood	Number According to Disease State	Suppressive Activity	Reference
CD4^+^CD25^+^FoxP3^+^	Decreased	Decreased	[86]
	Decreased	Unchanged	[87]
	Decreased	Not studied	[88]
	Decreased	Not studied	[89]
	Decreased; no correlation with disease activity	Not studied	[90]
	Increased	Not studied	[91]
	Increased	Unchanged	[92]
CD4^+^CD25^low^GITR^+^	Increased in patients with inactive disease	Unchanged	[90]
Helios^+^FoxP3^+^	Increased	Increased	[93,94]
Tr1 cells	Decreased	Not studied	[95]

**Table 2:** Pharmaceuticals appropriate for B-cell therapy for pSS.

Drug	Target	Mechanism	Efficacy	Reference
Rituximab	CD20 on B-cell surface	Chimeric anti-CD20 antibody. Causes antibody-dependent cellular cytotoxicity, complement-mediated cytotoxicity, and apoptosis-mediated transient depletion of B cells in peripheral blood, salivary glands, and other target tissues.	Depends on the dose and duration of therapy. With a well-chosen course, stimulation of salivation and improvement in the function of lacrimal glands, a decrease in the activity of the disease according to the ESSDAI, and a reduction in infiltrates and GCs.	[108,109,110,111,112,113,114,115]
Epratuzumab	CD22 on B-cell surface	Humanized anti-CD22 antibody. Modulates B-cell activity (CD22 regulates B-cell function via CD19 and B-cell antigen receptor (BCR) signaling and induces BCR-induced cell death. CD22 also regulates TLR signaling and controls B-cell survival in peripheral organs).	Significant improvement in the function of the lacrimal glands, unstimulated salivation, and elimination of symptoms of fatigue.	[116,117]
Belimumab	BAFF	Human monoclonal antibody. Inhibits BAFF, thereby preventing their activation and proliferation.	Decrease in parotid edema and levels of B-cell activation biomarkers. No change in unstimulated salivation or Schirmer test.	[118]
Ianalumab (VAY736)	BAFF	Inhibits BAFF, leading to blockade of BAFF-mediated signaling and deletion of B cells. Direct lysis of B cells via antibody-dependent cellular cytotoxicity.	Dose-dependent reduction in disease activity according to the ESSDAI.	[119]
Remibrutinib	BTK	Inhibits BTK on B cells, leading to impaired BCR signaling that regulates B-cell proliferation and survival.	Improvement in the ESSDAI, salivary flow, and pathologically elevated immunoglobulins as signatures of activity.	[120,121]
Baminercept	Lymphotoxin-β receptor (LTβR) on B-cell surface	Recombinant lymphotoxin-β receptor fusion protein. Blockade of LTβR-mediated signaling inhibits lymphocytic infiltration and formation of ectopic GCs.	There was no significant decrease in disease activity according to the ESSDAI, no significant improvement in the secretion of the salivary and lacrimal glands, and extraglandular manifestations. Significant changes in the number of circulating T and B lymphocytes.	[122]

**Table 3:** Pharmaceuticals appropriate for T-cell therapy for pSS.

Drug	Target	Mechanism	Efficacy	Reference
Abatacept	CD80/86 on APC surface	Blocks the interaction between CD80/86 on APC surface with CD28 of T-cell surface, which is important for proliferation of T lymphocytes and production of cytokines.	Reduction in inflammation in salivary glands, improved salivation, reduction in the number of cTfh and Tregs, no changes in the foci of lymphoplasmacytic infiltration, and reduction in GCs.	[123,124,125,126]
Alefacept	CD2 on T-cell surface	Binds to CD2, inhibiting the interaction between LFA-3 and CD2, preventing the activation of T lymphocytes	Dose-dependent depletion of CD4^+^ and CD8^+^ cells in psoriasis	[127]
CFZ533 (iscalimab)	CD40 on APC and B-cell surfaces	Binds to CD40, blocking the interaction of APCs and B cells with CD40L of T lymphocytes	In phase 2 of clinical trials	[107]

## Data Availability

Not applicable.
